# Effect of bilberry juice on indices of muscle damage and inflammation in runners completing a half-marathon: a randomised, placebo-controlled trial

**DOI:** 10.1186/s12970-018-0227-x

**Published:** 2018-05-02

**Authors:** Anthony Lynn, Samantha Garner, Nichola Nelson, Trevor N. Simper, Anna C. Hall, Mayur K. Ranchordas

**Affiliations:** 1 Food Group, Sheffield Business School, Sheffield Hallam University, S1 1WB, Sheffield, UK; 2 Academy of Sport and Physical Activity, Sheffield Hallam University, S10 2BP, Sheffield, UK

**Keywords:** Bilberry, Polyphenols, Muscle damage, Inflammation

## Abstract

**Background:**

Emerging evidence indicates that fruits rich in polyphenols may attenuate exercise-induced muscle damage and associated markers of inflammation and soreness. This study was conducted to determine whether bilberry juice (BJ), which is particularly rich in polyphenols, reduces markers of muscle damage in runners completing a half marathon.

**Methods:**

A total of 21 recreationally trained runners (age 30.9 ± 10.4 y; mass 71.6 ± 11.0 kg; M = 16; F = 5) were recruited to a single blind, randomised, placebo-controlled, parallel study. Participants were block randomised to consume 2 × 200 ml of BJ or energy-matched control drink (PLA) for 5 d before the Sheffield Half Marathon, on race day, and for 2 days post-race. Measurements of delayed onset muscle soreness (DOMS), muscle damage (creatine kinase; CK) and inflammation (c-reactive protein; CRP) were taken at baseline, pre-race, post-race, 24 h post-race and 48 h post-race. The effect of treatment on outcome measures was analysed using magnitude-based inferences based on data from 19 participants; 2 participants were excluded from the analyses because they did not provide samples for all time points.

**Results:**

The half marathon caused elevations in DOMS, CRP and CK. BJ had a *possibly harmful* effect on DOMS from pre-race to immediately post-race (11.6%, 90% CI ± 14.7%), a *likely harmful* effect on CRP from pre-race to 24 h post-race (mean difference ES 0.56, 90% CI ± 0.72) and a *possibly harmful* effect on CRP from pre-race to 48 h post-race (ES 0.12, 90% CI ± 0.69). At other time points, the differences between the BJ and PLA groups in DOMS and CRP were *unclear, possibly trivial* or *likely trivial*. Differences in the changes in CK between BJ and PLA were *unclear* at every time point other than from baseline to pre-race, where BJ had a *possibly harmful* effect on reducing muscle damage (ES 0.23, 90% CI ± 0.57).

**Conclusion:**

Despite being a rich source of antioxidant and anti-inflammatory phytochemicals, BJ evoked small to moderate increases in exercise-induced DOMS and CRP. Further larger studies are required to confirm these unexpected preliminary results.

## Background

Long distance running causes muscle damage which is characterised by a temporary loss of force production and delayed onset muscle soreness (DOMS) [1, 2]. The mechanisms underpinning these effects are not fully understood. One theory proposes that during long distance running, eccentric muscle actions disrupt muscle fibres and this disruption causes: (i) a loss of force and (ii) a secondary inflammatory response that produces the symptoms of DOMS [3]. Metabolic stress encountered during long distance running may also contribute to producing the symptoms of muscle damage [4]. Because inflammation and DOMS may impact on performance and the ability to train, the use of non-steroidal anti-inflammatory drugs (NSAIDs) is common among endurance athletes [5]. Although, NSAIDs may reduce inflammation and pain, their use is associated with a range of adverse effects such as gastrointestinal bleeding, renal and vascular problems [6]. NSAIDs may also interfere with the adaptive response to training, at least in young athletes [7]. Consequently, there is interest in identifying alternative approaches to reducing inflammation and promoting recovery.

Plant derived polyphenols exhibit anti-inflammatory effects and emerging evidence suggests they can reduce exercise-induced inflammation and soreness [2, 4, 8–14]. Tart cherry juice has been shown to promote recovery of muscle strength [2, 9–11], reduce markers of inflammation [2, 4, 8, 9, 14] and oxidative stress [2, 4, 10] and possibly ameliorate muscle soreness [9, 11, 15] in runners [2, 9, 14, 15], cyclists [4, 8] and weight trainers [10, 11]. Blueberries [13] and extracts/juice of pomegranate [12, 16] have also been demonstrated to promote the recovery of muscle strength after a bout of eccentric resistance exercise [12, 13], but the effect of other fruits rich in polyphenols remains underexplored.

Bilberries are a particularly rich source of polyphenolic compounds [17], but their effect on exercise-induced muscle damage (EIMD) and inflammation has not been reported. In cultured human colonic epithelial cells, extracts of bilberry inhibit the expression of pro-inflammatory genes [18]. The anti-inflammatory action of bilberries has also been confirmed in human intervention studies [19, 20]. Karlsen et al. [19] reported that in adults at elevated risk of cardiovascular disease, the consumption of 330 ml/d of bilberry juice (BJ) for 4 weeks reduced the concentrations of a range of markers of inflammation. Similarly, in adults with features of metabolic syndrome, the consumption of 400 g/d of whole bilberries for 8 weeks reduced an index of inflammation calculated from the sum of the Z scores for hsCRP, Interleukin-6 (IL-6), IL-12 and lipopolysaccharides [20]. Collectively, these results indicate that bilberries exert anti-inflammatory actions. So, this study was conducted to determine whether supplementation of the diet with BJ would reduce inflammation and associated muscle soreness in recreationally trained runners completing a half marathon.

## Methods

### Participants

A total of 21 recreationally trained runners (male *n* = 16; female *n* = 5) that had signed up to complete the 2013 Sheffield Half Marathon were recruited to an intervention study to determine whether BJ reduced post-exercise muscle damage, inflammation and soreness. The runners were recruited through personal contacts and targeted emails to running and triathlon clubs. Runners with a self-predicted finishing time of over 2 h were excluded. All participants completed health screening and physical activity readiness questionnaires to check for the presence of cardiovascular disease, hypertension, type 1 or type 2 diabetes, kidney disease, gastrointestinal problems, musculoskeletal injuries and food allergies. Participants also provided details of the length of time they had been running and the number of half and/or full marathons they had finished. The study was approved by the Faculty Ethics Committee of Sheffield Business School, Sheffield Hallam University (approval number SBSREC1213/13), UK and all participants provided written informed consent.

### Study design

**Fig. 1:**
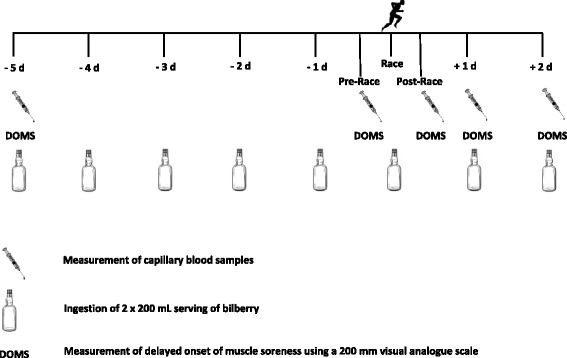
Experimental Protocol

The study was an 8 day, single-blind, placebo-controlled, parallel-group intervention of BJ versus a fruit flavoured maltodextrin drink (PLA). Participants were stratified by sex and then block randomised to either test drink (block size of 4). Test drinks (2 × 200 ml servings/d) were consumed for 5 days prior to the Sheffield Half Marathon, on the day of competition and for two days post-race. Participants were instructed to drink one serving of their test drink with breakfast and one with their evening meal. At baseline, pre-race, post-race, 1 d post-race and 2 d post-race muscle soreness was assessed and capillary blood samples were collected (Fig. 1). On race day, capillary blood samples and assessments of muscle soreness were collected at the race venue. Blood samples were then stored in a refrigerator in a motorhome before being transferred to the laboratory for processing. At other time points, samples were collected in the laboratory or at the homes of participants. All samples collected off-site were stored on ice for transportation to the laboratory. Except on race day, all samples were collected within a 4 h time window between 4 pm and 8 pm to suit the participants’ availability. For the duration of the study, participants completed a food diary. Participants were asked to avoid taking anti-inflammatory medication and antioxidant supplements during the study. At the start of the race, environmental conditions were: temperature 8.9 °C, wind speed 5 mph, barometric pressure 750 mmHg and humidity 73%.

### BJ and PLA drinks

Participants were provided with a supply of drinks at their first visit and instructed to store the drinks in a refrigerator. BJ was supplied by The Pure Juice Company, Twickenham, UK. Each 200 ml serving of juice provided 114 kcal, 29 g carbohydrate, 1.4 g protein, 0.6 g fat. The total phenol content of the BJ was determined using the Folin-Ciocalteau method and expressed as gallic acid equivalents [21] and the total anthocyanin content was measured by the pH differential method and expressed as cyanidin-3-glucoside equivalents [22]. All analyses were conducted in duplicate on three bottles of BJ.

The PLA drink was prepared from a fruit flavoured maltodextrin powder (Science in Sport, Go Energy, Nelson, Lancashire, UK) which was made up to a total volume of 200 ml with water. It was prepared to match the BJ for energy content. After preparation the drink was pasteurised by heating for 2 min at 75 °C in an industrial retort (Levati Food Tech, Traversetolo, Italy). The study was advertised to potential participants as a study on the effect of fruit flavoured drinks on recovery, so participants were unaware which drink was the experimental drink.

### Measurement of muscle soreness

Participants rated their lower body muscle soreness on a 200 mm visual analogue scale anchored at one end with ‘no pain’ and the other end with ‘unbearable pain’. Participants were asked to squat down to a 90^o^ angle and return to a standing position before rating their lower body muscle soreness [2].

### Blood collection and biochemical analyses

Capillary blood samples were collected into serum separator microvettes (Sarstedt, Leicester, UK) and allowed to clot for at least 30 min. Samples were then centrifuged at 10, 000 g × 5 min and serum was removed, divided into aliquots and stored at -80 °C until analysis.

Creatine kinase was determined in duplicate using a commercial enzyme kinetics kit (Spinreact, Girona, Spain) on a Cecil Aquarius U*V*/VIS spectrophotometer (Cecil Instruments, Cambridge, UK). The intra and inter assay CVs reported by the manufacturer were 0.84% and 2.01% [23].

Serum CRP was determined in duplicate using a commercial enzyme-linked immunosorbent assay (R&D Systems, Abingdon, UK). Accuracy was assessed using an external quality control; European Reference Material DA472/IFCC. The certified value was 41.8 mg/L; mean of assay was 42.8 ± 2.99) mg/L; CV 6.98*%; n* = 8.

### Statistical analysis

The effect of the intervention on each outcome marker was analysed using magnitude-based inferences [24]. Body mass was entered as a covariate into all analyses to account for an imbalance in mean body mass between groups at baseline. Prior to analysis, the DOMS data were converted into percentages and the CRP and CK data were log transformed [25]. The uncertainty in the likelihood that the true (population) effect of BJ was substantially harmful or beneficial was expressed as 90% confidence intervals. The thresholds for benefit or harm were: ± 10% for DOMS and a Cohen’s d of ±0.2 for CRP and CK [25]. The between-subject standard deviations were used to convert the log transformed changes in CRP and CK into Cohen’s d values [26]. If the confidence interval for an effect overlapped substantial benefit and harm then effects were deemed unclear. Other effects were declared beneficial, trivial or harmful using the following criteria: < 0.5% = almost certainly not; < 5% = very unlikely; < 25% = unlikely; 25–75% = possibly; > 75% = likely; > 95% = very likely; > 99.5% = almost certainly [27]. All analyses were conducted using a freely available published spreadsheet [27].

## Results

### Characterisation of the bilberry juice

The total phenol content per 200 ml serving of BJ was 744.14 ± 81.75 mg (*n* = 3). Each 200 ml bottle also contained 80.04 ± 3.51 mg of total anthocyanins (*n* = 3).

### Participant characteristics

The characteristics of the participants are reported in Table 1. The mean finishing time in both groups was similar suggesting that the groups were well matched in respect to performance ability.

**Table 1:** Characteristics of the half-marathon runners in the BJ and PLA groups

	BJ	PLA
Age (y)	31.4 ± 8.7	30.5 ± 12.37
Sex (M/F)	8/3	8/2
Height (m)	1.75 ± 0.11	1.73 ± 0.06
Mass (kg)	74.3 ± 13.1	68.6 ± 7.9
BMI (kg/m^2^)	24.2 ± 3.0	22.8 ± 1.9
Finishing time (h:min:s)	1:41:06 ± 0:14:24	1:38:02 ± 0:10:17
Previous full and ½ marathons completed	2 (0.25, 8.75)	1 (0.25, 2.0)
Duration of running experience (y)	3.5 (1.3, 8.8)	2 (1.5, 7.0)

Values are mean ± SD, except for previous full and ½ marathons and duration of running experience which are expressed as median and interquartile range; *n* = 11 in BJ group; *n* = 10 in PLA group, except for history of previous marathons and running experience where *n* = 10 for both groups.

### Retention and compliance

Of the 21 runners that enrolled onto the study, data was collected from 19 at all the study’s time points. Of the remaining two runners (both in the BJ group), one female runner failed to report to the mobile laboratory immediately after the race and one male runner failed to provide a sample on the final day of the study; their data were removed from the statistical analyses. There were no reports of adverse effects to the BJ or PLA and all runners reported consuming their test drinks.

### Inflammation (C-reactive protein)

**Fig. 2:**
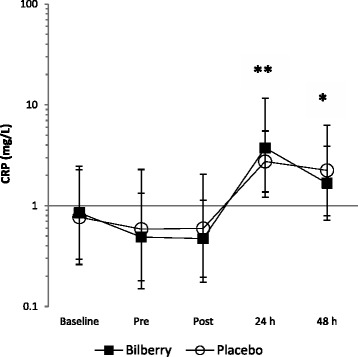
Change in CRP. Values are geometric means (GM) and the error bars represent ×/÷ SD of the GM [35];* *possibly harmful* greater increase in CRP from pre-race to 48 h post-race ** *likely harmful* greater increase in CRP from pre-race to 24 h in BJ group relative to PLA group (BJ group *n* = 9; PLA group *n* = 10)

From baseline to pre-race there was an *unclear* effect of BJ on CRP relative to PLA (mean difference ES -0.15, 90% CI ± 0.54). There was a *likely trivial* difference between groups in the change in CRP from pre-race to post race (mean difference ES -0.03, 90% CI ± 0.25). BJ evoked a moderately larger, *likely harmful* increase in CRP from pre-race to 24 h post-race than PLA (mean difference ES 0.56, 90% CI ± 0.72). One participant in the BJ group had a vastly elevated CRP at 24 post-race. Removal of their CRP value slightly attenuated the ES from 0.56, 90% CI ± 0.72 to 0.50, 90% CI ± 0.72, but the effect of BJ remained *likely harmful*. The difference in the change in CRP from pre-race to 48 h post-race between BJ and PLA was *possibly harmful* (mean difference ES 0.12, 90% CI ± 0.69) (see Fig. 2).

### Muscle soreness

**Fig. 3:**
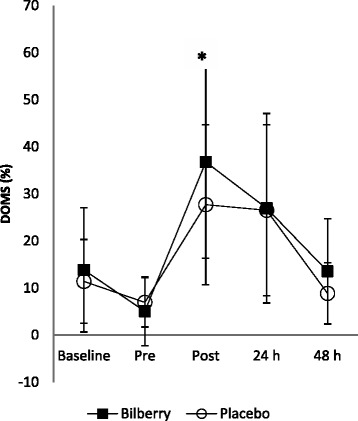
Change in reported muscle soreness. Values are displayed as mean percentages and error bars are ±SD * *possibly harmful* greater increase in DOMS from pre-race to immediately post-race in BJ group relative to PLA (BJ group n = 9; PLA group n = 10)

There was a small fall in reported muscle soreness from baseline to pre-race. The decrease was slightly larger in the BJ group than in the PLA group (mean difference − 5.9%, 90% CI ± 7.8%; *likely trivial*). The half-marathon evoked a substantial increase in reported muscle soreness which peaked immediately post-race. There was a small *possibly harmful* greater increase in muscle soreness from pre-race to immediately post-race in the BJ group than in the PLA group (11.6%, 90% CI ± 14.7%). From pre-race to 24 h post-race, the difference in the change in reported muscle soreness between groups was *possibly trivial* (mean difference − 0.1%, 90% CI ± 15.7%), whereas from pre-race to 48 h post-race, the difference was *likely trivial* (mean difference 6.9%, 90% CI ± 5.7%) (see Fig. 3).

### Muscle damage (Creatine kinase)

**Fig. 4:**
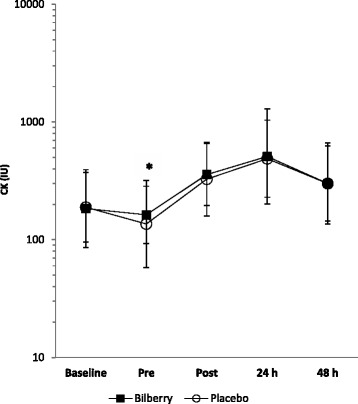
Change in creatine kinase. Values are geometric means (GM). The error bars represent the ×/÷ SD of the GM [35] * *possibly harmful* smaller decrease in CK from baseline to pre-race in BJ group relative to PLA (BJ group n = 9; PLA group n = 10)

Creatine kinase, an indirect marker of muscle damage fell from baseline to pre-race in both groups, but the decrease was smaller in the BJ group than the PLA group, indicating a *possibly harmful* effect of BJ (mean difference ES 0.23, 90% CI ± 0.57). CK rose in response to the half-marathon peaking at 24 h post-race. There were unclear differences in CK responses to the half-marathon between the BJ group and the PLA group from pre-race to post-race (mean difference ES 0.02, 90% CI ± 0.49), from pre-race to 24 h post-race (mean difference ES -0.10 90% CI ± 0.67), and from pre-race to 48 h post-race (mean difference ES -0.14, 90% CI ± 0.52) (see Fig. 4).

## Discussion

The present study found that a half marathon elicited substantial elevations in DOMS and markers of muscle damage (CK) and inflammation (CRP). This suggests that in recreationally trained runners, a half-marathon is an appropriate exercise challenge for investigating the effect of a dietary intervention on protection against EIMD and promotion of recovery.

Immediately after the race, there was evidence of a *possibly harmful* effect of BJ on DOMS in comparison to PLA. The *possibly harmful* effect of BJ on post-race DOMS was mirrored by a *likely harmful* effect on CRP at 24 h post-race and a *possibly harmful* effect at 48 h post-race. This dual rise in DOMS and CRP indicates that runners consuming the BJ may have suffered more muscle damage during the half-marathon than those consuming the energy-matched PLA drink, although this was not reflected by a clear difference between groups in the change in CK from pre-race to any time point post-race. The lack of a clear difference in CK between groups may be partly explained by the large differences in CK response among our runners and the small sample size. Large inter-individual variability in the release of CK from muscles in response to protocols designed to induce muscle damage has been widely reported in the literature [28, 29]. Moreover, evidence indicates that the degree of elevation in circulating CK may not directly correlate with the extent of muscle damage or loss of force [30, 31]. Therefore, circulating CK may not be a particularly sensitive marker for detecting small differences between individuals in the extent of muscle damage.

Our findings of a small, *possibly harmful* increase in DOMS immediately post-race and a *likely harmful* moderate increase in CRP at 24 h post-race were unexpected, because we originally hypothesised that the polyphenols in BJ would exhibit anti-inflammatory effects and possibly protect muscles against soreness. To our knowledge no other studies have reported on the effects of BJ on EIMD, however, our results contrast with evidence that juice and extracts of tart cherry protect against inflammation induced by long distance running [2, 14], intermittent running [9], and high intensity cycling [4, 8]. The effect of tart cherry beverages/supplements on DOMS is mixed with some studies reporting no effect [2, 8] and others reporting a reduction [9, 14, 15], although we are unaware of any studies that have reported an increase. The disagreement between our study and the studies of tart cherry is difficult to explain, but could reflect differences in the quantity and type of polyphenols present in the juices. In-house analysis of the BJ revealed that our dosing strategy seemed to supply greater quantities of total phenols (≈1500 mg/d) and anthocyanins (≈160 mg/d) than the tart cherry supplementation studies in runners (ranges reported in the literature; total phenols 991–1200 mg/d; total anthocyanin 66–80 mg/d) [2, 14, 15]. Although, we originally hypothesised that a high intake of polyphenols might protect against exercise-induced inflammation and soreness, it is not inconceivable that it could produce the opposite effect. The production of reactive oxygen species (ROS) by exercising muscles has been proposed to act as a brake on muscle contraction thereby limiting muscle damage during prolonged periods of contraction [32]. BJ polyphenols may have enhanced intramuscular antioxidant protection to the extent that it reduced the braking effect of ROS on muscle contraction thus enabling the participants to run harder, but at the expense of generating more muscle damage, soreness and inflammation [32]. We are unaware of any studies reporting improved performance coupled with increases in markers of soreness or inflammation in response to supplementation with polyphenols, however, Cobley et al. [33] found that acute supplementation with the antioxidant N-acetylcysteine improved performance, but elevated muscle damage in recreationally trained runners completing the Yo-Yo Intermittent Recovery Test after a muscle damaging intermittent shuttle run test. Unexpected effects of antioxidant micronutrients on muscle recovery have also been reported [34]. Close et al. [34] found that supplementation with 1 g of ascorbic acid 2 h before and daily for 14 days after downhill running delayed the recovery of muscle function.

### Limitations

This study has several limitations. First, we did not measure any functional markers of muscle strength, thus it is impossible to determine whether the small increase in DOMS and moderate increase in CRP that we observed resulted in a greater loss of muscle force or slower recovery of muscle strength. Second, the timing of the assessment of muscle soreness after the race varied slightly between runners because they had to make their way through a busy finishing area to our mobile laboratory for assessment. It is possible that this small variation in time lapse (approximately 5 min) may have influenced the post-race assessment of DOMS. Third, we only followed our participants for 2 days after the half marathon. Whilst this is consistent with two studies of tart cherry and long distance running [2, 15], it is possible that 2 days may have been insufficient to fully capture the effects of BJ on CRP and CK both of which had not returned to pre-race levels at the end of the study. Fourth, except for prohibiting the use of antioxidant vitamins and NSAIDs, we placed no restrictions on the diet of our participants. We asked participants to complete a food diary for the duration of the race, but a number in both groups failed to complete their diaries so we were unable to accurately assess whether runners in either group changed their diet in any way that may have substantially influenced our results. It is possible that standardising the diets of the participants and restricting their intake of foods rich in polyphenols may have enhanced our ability to detect more effects of the BJ intervention. Fifth, the use of capillary blood sampling techniques limited the number of biomarkers that we could measure. The collection of larger blood samples would have enabled us to measure a more comprehensive battery of inflammatory markers and a range of markers of oxidative stress, which may have proved informative. Sixth, we adjusted the effects of our intervention for body mass, but this may not have adequately accounted for differences in lean and fat mass, which could have contributed to the observed effects. Finally, whilst our sample size was similar to comparable studies, the statistical analysis revealed a number of *unclear* outcomes indicating that a greater sample size was needed to increase the precision of our estimates of treatment effects.

## Conclusion

The present study provides preliminary evidence that consuming BJ for 5 days before and 2 days after a half-marathon may evoke small to moderate transient increases in muscle soreness and inflammation in recreationally trained runners. Our surprising results require confirmation in a larger study, which should also determine whether the transient detrimental changes we observed result in adverse functional changes in muscle strength and recovery.

## Abbreviations

BJ: Bilberry juice
BMI: Body mass index
CI: Confidence interval
CK: Creatine kinase
CRP: C-reactive protein
CV: Correlation co-efficient
DOMS: Delayed onset muscle soreness
EIMD: Exercise-induced muscle damage
ES: Effect size
g: Gravitational force
IQR: Interquartile range
kg: Kilograms
mg: Milligrams
mmHg: Millimetres of mercury
mph: Miles per hour
NSAIDs: Non-steroidal anti-inflammatory drugs
PLA: Placebo
ROS: Reactive oxygen species
SD: Standard deviation **y**: Years
